# Effects of Plyometric Jump Training in Female Soccer Player’s Physical Fitness: A Systematic Review with Meta-Analysis

**DOI:** 10.3390/ijerph17238911

**Published:** 2020-11-30

**Authors:** Mario Sánchez, Javier Sanchez-Sanchez, Fabio Y. Nakamura, Filipe M. Clemente, Blanca Romero-Moraleda, Rodrigo Ramirez-Campillo

**Affiliations:** 1 Research Group Planning and Assessment of Training and Athletic Performance, Universidad Pontificia de Salamanca, 37002 Salamanca, Spain; msanchezga@upsa.es (M.S.); jsanchezsa@upsa.es (J.S.-S.); 2 Associate Graduate Program in Physical Education, Universidade Federal do Paraiba, Joao Pessoa, PB 58051-970, Brazil; fabioy_nakamura@yahoo.com.br; 3 Escola Superior Desporto e Lazer, Instituto Politécnico de Viana do Castelo, Rua Escola Industrial e Comercial de Nun’Álvares, 4900-347 Viana do Castelo, Portugal; Filipe.clemente5@gmail.com; 4 Instituto de Telecomunicações, Delegação da Covilhã, 1049-001 Lisboa, Portugal; 5 Department of Physical Education, Sport and Human Movement, Universidad Autónoma de Madrid, 28049 Madrid, Spain; blanca.romero@uam.es; 6 Applied Biomechanics and Sports Technology Research Group, Autonomous University of Madrid, 28049 Madrid, Spain; 7 Human Performance Laboratory, Department of Physical Activity Sciences, Universidad de Los Lagos, Osorno 5290000, Chile; 8 Centro de Investigación en Fisiología del Ejercicio, Facultad de Ciencias, Universidad Mayor, Santiago 7500000, Chile

**Keywords:** human physical conditioning, resistance training, exercise therapy, plyometric exercise, football, sports, athletic performance

## Abstract

We aimed to assess the effects of plyometric jump training (PJT) on female soccer player’s physical fitness. To this aim, a systematic review with meta-analysis (SRMA) was conducted. The electronic databases PubMed, MEDLINE, Web of Science, and SCOPUS were used. To qualify for inclusion, peer-reviewed studies must have included (i) a PJT programme of ≥2 weeks, (ii) healthy athletes, (iii) a control group, and (iv) physical fitness outcomes (e.g., jump; sprint). Studies were excluded if (i) they incorporated injuried female soccer players, (ii) did not involve PJT or an active control group, (iv) lack of baseline and/or follow-up data. Data was meta-analyzed using the inverse variance random-effects model. Ten moderate-to-high quality studies were included in the analyses, comprising 13 training groups (*n* = 140) and 10 control groups (*n* = 110). Small to large (ES = 0.60–2.24; *p* = 0.040 to <0.001) effects were noted for countermovement jump, drop jump, kicking performance, linear sprint, change of direction speed, and endurance. The moderator analyses (i.e., PJT duration, age groups, competitive level, and soccer experience) revealed no significant differences between groups. In conclusion, PJT may improve the physical fitness of female soccer players. Such improvements might be expected after PJT interventions with six or more weeks of duration, and in players with different chronological ages, competitive levels and soccer experience.

## 1. Introduction

Endurance (cardiorespiratory capacity) is a relevant physical fitness trait in soccer [1]. However, jumping, single and repeated sprinting, change of direction, and kicking are also key proxies of soccer performance [2,3,4]. Indeed, these maximal-intensity single-effort physical fitness traits preceded goal opportunities in competitive leagues [2,5]. For example, sprinting and jumping actions occur in more than 50% of goal situations [2]. Moreover, mean values of acceleration >2.26 m·s^−2^ occur 1.78 times per minute, in addition to sprint distance of 2.87 m per minute, and/or highs-peed running of 6 meter per minute, with greater values during highly-competitive matches [6]. In addition, maximal-intensity short-duration actions such as jumping and sprinting may be associated with team position in a given tournament and/or players’ competitive level [4,7,8]. 

Several training approaches are used among female soccer players to improve jumping, single and repeated sprinting, change of direction and kicking power, as well as endurance attributes [5]. However, plyometric jump training (PJT) may be particularly effective, offering several advantages (e.g., reduced cost; injury prevention) compared to other methods (e.g., traditional resistance training) [9,10]. Additionally, the incorporation of PJT among training practices in soccer might be highly translated into game scenarios. For instance, there is a strong reliance on vertical and horizontal expressions of power during various game scenarios in soccer such as when defending, shooting, and attacking [1,5,11]. In turn, according to the principle of training specificity, soccer players should regularly engage in PJT programs. Indeed, PJT have demonstrated a significant transference effect between jump training exercises and soccer-specific physical performance [12,13,14]. 

From a physiological perspective, PJT capitalizes on the stretch-shortening cycle (SSC) where musculotendinous units are eccentrically stretched during the loading or impact phase before concentrically shortening in the push-off or take-off [15,16]. In this regard, PJT results in a wide range of distinct physiological and biomechanical adaptations (e.g., increased motor unit recruitment and rate of force development (RFD) [17,18,19,20]. Among soccer athletes (mixed with other sports), after 8 weeks of PJT, significant changes were noted at the muscle fiber level, including myosin heavy chain isoform composition in type I/IIa fibers, increased cross-sectional area, absolute peak calcium-activated force, maximum unloaded and loaded shortening velocity, absolute and normalized peak power, velocity at peak power, absolute force at which peak power was reached, and increased stiffness [21]. In a similar study, increased percentage of type I/IIa and IIa fibers were noted, in line with a decrease in IIx fibres from the vastus lateralis [22]. In another study with soccer players, 8 weeks of PJT combined with resistance training increased electromyography activity (72–110%) in the vastus medialis and rectus femoris muscles during jumping [23]. Another study with soccer players reported significant increases (4–15%; ES = 0.3–1.3) in leg stiffness after 4 weeks of training [24].

Due to the beneficial effects of PJT, several systematic reviews and meta-analysis (SRMA) have been published evidencing the effectiveness of this training mode to improve distinct power-related attributes in athletes from different sports disciplines including handball [25] and volleyball [26]. Likewise, there is a growing body of experimental evidence examining the effects of PJT on physical fitness attributes in female soccer players [27]; however, this evidence has not yet been comprehensively aggregated. Although a recent SRMA examined the effects of PJT on female soccer, only vertical jump height (i.e., countermovement jump) was analyzed [28]. Another SRMA examined the effects of PJT on male soccer players physical fitness [29], including measures such as jumping, sprinting and strength. However, considering the differences between female and male soccer players [1,5] and their potential different response to PJT [30], it would be adventurous to extrapolate results derived from male to female soccer players. 

Given the increased scientific awareness of PJT relevance regarding physical fitness improvement, it was deemed appropriate to aggregate PJT studies conducted in female soccer players in a SRMA to strengthen the level of scientific evidence [31] on this topic. This knowledge can guide practitioners to use PJT routines that are effective in female soccer, avoiding the simple transfer from the knowledge related to male soccer players. Thus, the aim of this SRMA was to assess the effects of PJT on female soccer player’s physical fitness (e.g., jump, sprint, kicking ability, change of direction speed; anaerobic performance; endurance).

## 2. Materials and Methods 

This SRMA was conducted and reported in accordance with the PRISMA statement [32].

### 2.1. Eligibility Criteria

A Participants, Intervention, Comparators, Outcomes, and Study (PICOS) design approach was used to rate studies for eligibility [32]. The respective inclusion criteria adopted in our meta-analysis was as follow: (i) apparently healthy female soccer players, with no restrictions on their playing level or age, (ii) a PJT programme, defined as lower-body unilateral or bilateral bounds, jumps, and hops that commonly utilise a pre-stretch or countermovement stressing the stretch-shortening cycle, (iii) a control group, (iv) at least one measure of physical fitness (e.g., jump, sprint, kicking ability, change of direction speed; anaerobic performance; endurance) before and after PJT, (v) controlled trials. The respective exclusion criteria adopted in our meta-analysis was as follow: (i) female soccer players with health problems (e.g., injuries, recent surgery), (ii) exercise interventions not involving PJT or exercise interventions involving PJT programmes representing less than 50% of the total training load when delivered in conjunction with other training interventions (e.g., high-load resistance training), (iii) absence of active control group, (iv) lack of baseline and/or follow-up data, (v) non-controlled trials. Of note, two previous scoping reviews [27,33] indicated that several otherwise high-quality studies in the field of PJT did not include a randomization design. In order to avoid the exclusion of potentially relevant studies, we considered for inclusion non-randomized study designs, as long as baseline values between groups were similar for the main outcome of the study. In contrast, the inclusion of an active control group was considered essential in order to isolate the effect of PJT from the rest of training methods that female soccer players commonly conduct in their regular training schedule.

Additionally, only full-text, peer-reviewed, original studies written in English were considered, excluding cross-sectional, review papers, or training-related studies that did not focus on the effects of PJT exercises (e.g., studies examining the effects of upper-body plyometric exercises). Retrospective studies, prospective studies, studies in which the use of jump exercises was not clearly described, studies for which only the abstract was available, case reports, special communications, letters to the editor, invited commentaries, errata, overtraining studies, and detraining studies were also excluded from the present meta-analysis. In the case of detraining studies, if there was a training period prior to a detraining period, the study was considered for inclusion.

### 2.2. Information Sources

We considered previous recommendations from the two largest scoping reviews examining PJT to conduct the literature search [27,33]. Computerized literature searches were conducted in the electronic databases PubMed (comprising MEDLINE), Web of Science Core Collection, and SCOPUS. The search strategy was conducted using the Boolean operators AND as well as OR with the following keywords: “ballistic”, “complex”, “explosive”, “force-velocity”, “plyometric”, “stretch-shortening cycle”, “jump”, “training”, “female”, “women”, “football”, and “soccer”. For example, the following search was adopted using Pubmed: (((((((((“randomized controlled trial” [Publication Type]) OR “controlled clinical trial” [Publication Type]) OR “randomized” [Title/Abstract]) OR “trial” [Title]) OR “clinical trials as topic” [MeSH Major Topic]) AND “soccer” [Title/Abstract]) OR “football” [Title/Abstract]) AND “training” [Title/Abstract]) OR “plyometric” [Title/Abstract]). After an initial search (April 2017), accounts were created in the respective databases. Through these accounts, automatically generated emails were received for updates regarding the search terms used. The search was refined in May 2019 and updates were received daily (if available), and studies were eligible for inclusion up to January 2020. Following the formal systematic searches, we conducted additional hand-searches (e.g., personal libraries). One author (RRC) performed the only search. 

### 2.3. Study Selection and Data Collection Process

After the exclusion of repeated article titles, a review of retrieved article titles was conducted. Then, examination of article abstracts followed. Thereafter, full articles were assessed. The reasons to exclude full-text articles were recorded. The data extracted from the included articles was recorded in a pre-form created in Microsoft Excel (Microsoft Corporation, Redmond, WA, USA). Two authors (MSG and JSS) conducted the process independently, and a third author (RRC) resolved disagreements regarding study eligibility.

### 2.4. Data Items

For the current review, physical fitness was chosen as the main outcome. *A priori*, common measures of physical fitness were considered, but not limited to: (i) jump (i.e., height; distance; flight time; power; reactive strength [i.e., mm.ms; ms.ms]), (ii) sprint (i.e., time; velocity), (iii) kicking ability (i.e., distance; velocity), (iv) change of direction speed (i.e., time; speed), (v) anaerobic performance (e.g., repeated sprint ability mean time; 30-s Wingate test mean power), (vi) endurance (e.g., shuttle-run total time or distance). 

In addition to the aforementioned data items, adverse effects were registered, and descriptive characteristics of the PJT interventions (e.g., duration; frequency) and athletes (e.g., age; fitness level) were extracted. A complete description of the additional PJT and athletes’ characteristics have been previously published [33].

### 2.5. Methodological Quality in Individual Studies

The methodological quality of eligible studies was assessed using the Physiotherapy Evidence Database (PEDro) scale, as previously described [34] and interpreted for PJT literature [28,35]. Briefly, studies with ≤3 points were considered of poor quality, 4–5 points as moderate quality, and 6–10 points as high quality. Two of the authors (RRC and MSG) independently scored the articles. Disagreements in the rating between both authors was resolved through discussion with a third author (JSS). Aiming to control the risk of bias between authors, the Kappa correlation test was used to analyse the agreement level for the included studies. An agreement level of k = 0.89 was obtained. 

### 2.6. Summary Measures and Synthesis of Results, and Publication Bias

For analysis and interpretation of results, we followed previous recommendations for PJT meta-analyses [26,28,36,37]. Briefly, using a random-effects model meta-analyses were conducted only if ≥3 studies provided means and standard deviations for the same pre-post PJT parameter (e.g., sprint time), in order to calculate an effect size (ES; Hedges’ g ES) alongside their respective 95% confidence intervals (CIs). Data was standardized using post-intervention standard deviation score. Calculated ES were interpreted as previously recommended for sport sciences studies: <0.2, trivial; 0.2–0.6, small; >0.6–1.2, moderate; >1.2–2.0, large; >2.0–4.0, very large; >4.0, extremely large [38]. For studies that incorporated ≥2 intervention groups and only one control group, the sample size in the control group was proportionately divided during analyses [39]. Heterogeneity was assessed using the *I*^2^ statistic, with values of <25%, 25–75%, and >75% considered as representative of low, moderate and high heterogeneity, respectively. The risk of bias was analyzed with the Egger’s test [40]. To adjust for publication bias, a sensitivity analysis was conducted using the trim and fill method [41], with L0 as the default estimator for the number of missing studies [42]. Subgroup analyses to assess the potential influence of PJT duration (number of weeks), training frequency (number of sessions per week), total number of training sessions, in addition to the age, the expertise level of the participants (i.e., moderate-level vs. high-level players), and the athletes years of soccer experience were performed according to the median split technique. Analyses were performed using specialized software (Comprehensive Meta-Analysis; version 2; Biostat, Englewood, NJ, USA). Statistical significance was set at *p* < 0.05.

## 3. Results

### 3.1. Study Selection

**Figure 1:**
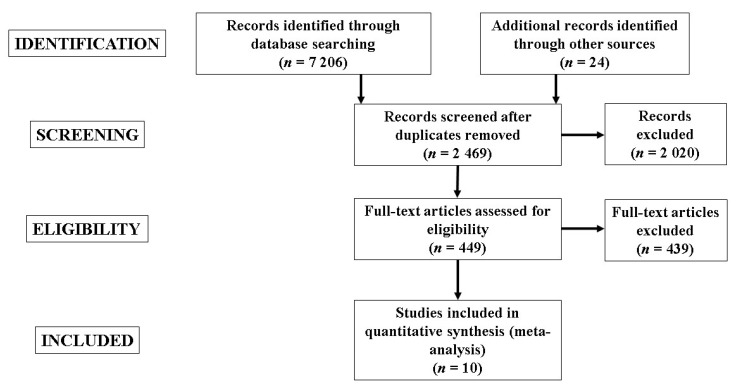
Flow diagram of the search process.

The electronic search process identified 7206 studies (2261 from PUBMED, 2280 from SCOPUS, and 2665 from WOS), plus 24 studies through other sources. Duplicate studies were then removed (n = 4761). Study titles and abstracts were screened with a further 2020 studies removed. Accordingly, full-text versions of 449 studies were screened. From these, 224 studies did not include an appropriate study design (e.g., control group), 188 studies did not include soccer players or female soccer players only, and 27 were excluded for different reasons (e.g., no measure of physical fitness provided). The remaining 10 studies [43,44,45,46,47,48,49,50,51,52] were included in the SRMA (Figure 1). The included studies involved 13 individual experimental groups and 140 participants, and 110 participants in the 10 control groups. The characteristics of the participants and PJT interventions are indicated in the Table 1 and Table 2. Briefly, the age of the participants was between a mean of 13.4 to 26.6 years, with a fitness level that varied from recreationally trained to professional athletes. The PJT interventions lasted between six up to 12 weeks. Almost all studies (except one) incorporated PJT during the in-season period. A complete description of the physical fitness measures used in the meta-analyses is provided in Table 3.

### 3.2. Methodological Quality

Using the PEDro checklist, five studies achieved 4 or 5 points and were classified as being of “moderate” quality, while five studies achieved 6–10 points and were therefore considered as being of “high” methodological quality (Table 4). 

### 3.3. Meta-Analysis Results for Countermovement Jump

**Figure 2:**
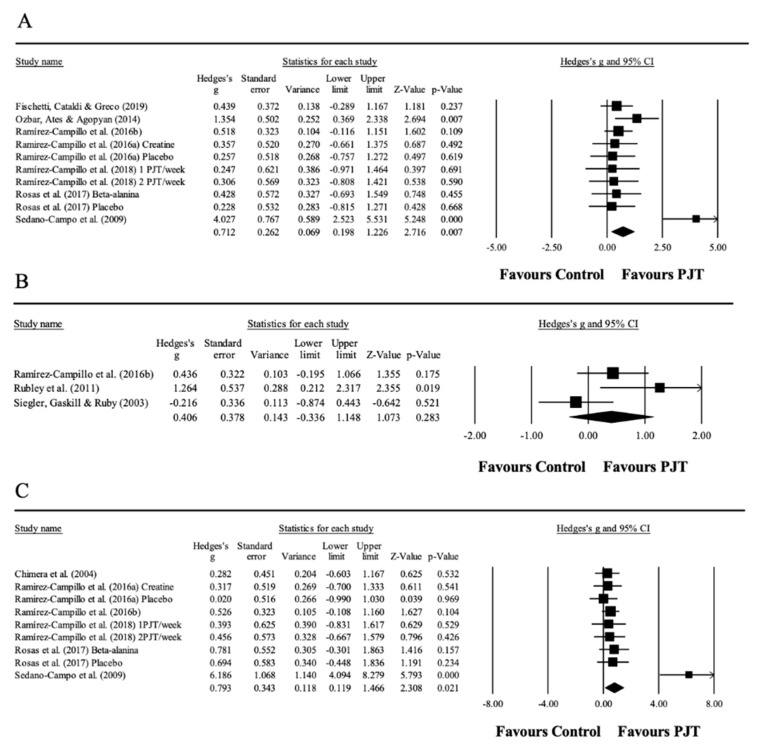
Forest plot of changes in countermovement jump (**A**), countermovement jump with arms awing (**B**), and drop jump performance (**C**) in athletes participating in plyometric jump training (PJT) compared to controls. Values shown are effect sizes (Hedges’s g) with 95% confidence intervals (CI). The size of the plotted squares reflects the statistical weight of the study.

Seven studies provided data for CMJ, involving 10 experimental and seven control groups (pooled *n* = 182). There was a significant effect of PJT on CMJ (ES = 0.71; 95% CI = 0.20 to 1.23; *p =* 0.007; *I*^2^ = 62.9%; Egger’s test *p* = 0.224; Figure 2A). The relative weight of each study in the analysis ranged from 6.9% to 13.3%.

No significant sub-group difference (between-group *p =* 0.188) was found when PJT interventions with ≤6 weeks (5 study groups; ES = 0.40; 95% CI = −0.01 to 0.80; within-group *I*^2^ = 0.0%) were compared to PJT interventions with >6 weeks (5 study groups; ES = 1.18; 95% CI = 0.09 to 2.28; within-group *I*^2^ = 81.1%). 

Similarly, no significant sub-group difference (between-group *p =* 0.664) was found when PJT interventions with players ≤22.8 years old (4 study groups; ES = 0.62; 95% CI = 0.17 to 1.07; within-group *I*^2^ = 0.0%) were compared to PJT interventions with players >22.8 years old (6 study groups; ES = 0.84; 95% CI = −0.04 to 1.71; within-group *I*^2^ = 76.6%). 

Moreover, no significant sub-group difference (between-group *p =* 0.080) was found when PJT interventions with high-level players (4 study groups; ES = 1.41; 95% CI = 0.26 to 2.56; within-group *I*^2^ = 85.3%) were compared to PJT interventions with moderate-level players (6 study groups; ES = 0.30; 95% CI = −0.14 to 0.75; within-group *I*^2^ = 0.0%). 

Furthermore, no significant sub-group difference (between-group *p =* 0.420) was found when PJT interventions conducted on players with ≤5.7 years of soccer experience (4 study groups; ES = 1.42; 95% CI = −0.05 to 2.89; within-group *I*^2^ = 83.8%) were compared to PJT interventions conducted on players with >5.7 years of soccer experience (5 study groups; ES = 0.40; 95% CI = −0.01 to 0.80; within-group *I*^2^ = 0.0%). 

### 3.4. Meta-Analysis Results for Countermovement Jump with Arm Swing

Three studies provided data for CMJA, involving three experimental and three control groups (pooled *n* = 88). There was a non-significant effect of PJT on CMJA (ES = 0.41; 95% CI = −0.34 to 1.15; *p* = 0.28; *I*^2^ = 65.3%; Egger’s test *p* = 0.452; Figure 2B). The relative weight of each study in the analysis ranged from 25.4% to 37.8%. 

### 3.5. Meta-Analysis Results for Drop Jump

Six studies provided data for DJ, involving nine experimental and six control groups (pooled *n* = 154). There was a significant effect of PJT on DJ (ES = 0.79; 95% CI = 0.12 to 1.47; *p =* 0.021; *I*^2^ = 73.1%; Egger’s test *p* = 0.063; Figure 2C). The relative weight of each study in the analysis ranged from 6.3% to 14.0%. 

### 3.6. Meta-Analysis Results for Kicking Performance

**Figure 3:**
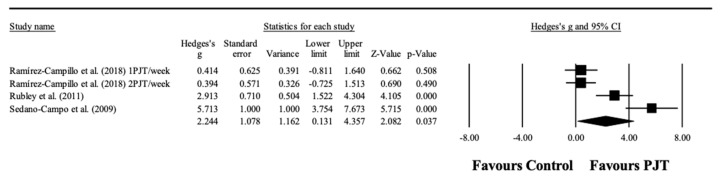
Forest plot of changes in kicking performance, in athletes participating in plyometric jump training (PJT) compared to controls. Values shown are effect sizes (Hedges’s g) with 95% confidence intervals (CI). The size of the plotted squares reflects the statistical weight of the study.

Three studies provided data for kicking performance, involving four experimental and three control groups (pooled *n* = 59). There was a significant effect of PJT on kicking performance (ES = 2.24; 95% CI = 0.13 to 4.36; *p =* 0.037; *I*^2^ = 89.4%; Egger’s test *p* = 0.040; Figure 3). After the trim and fill method, the adjusted values remained as the observed values. The relative weight of each study in the analysis ranged from 22.8% to 26.2%. 

### 3.7. Meta-Analysis Results for Linear Sprint

**Figure 4:**
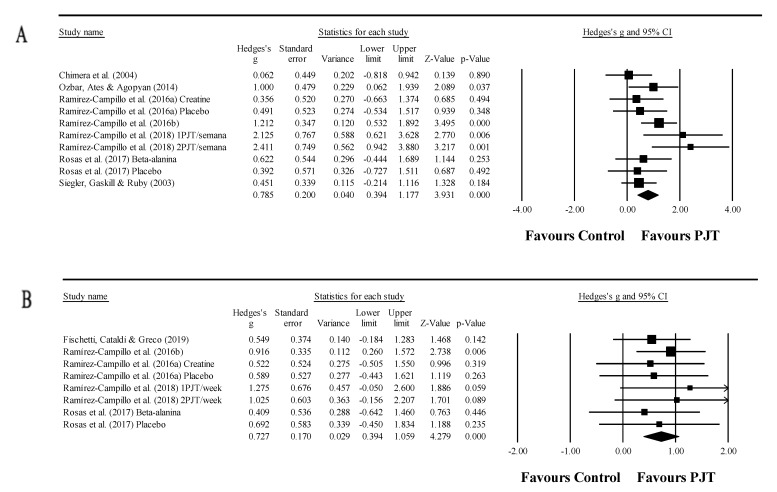
Forest plot of changes in linear sprint performance (**A**) (**upper**) and change of direction speed performance (**B**) (**bottom**), in athletes participating in plyometric jump training (PJT) compared to controls. Values shown are effect sizes (Hedges’s g) with 95% confidence intervals (CI). The size of the plotted squares reflects the statistical weight of the study.

Seven studies provided data for linear sprint performance, involving 10 experimental and seven control groups (pooled *n* = 186). There was a significant effect of PJT on linear sprint performance (ES = 0.79; 95% CI = 0.39 to 1.18; *p <* 0.001; *I*^2^ = 38.2%; Egger’s test *p* = 0.257; Figure 4A). The relative weight of each study in the analysis ranged from 5.4% to 15.3%. 

No significant sub-group difference (between-group *p =* 0.167) was found when PJT interventions with ≤6 weeks (six study groups; ES = 0.62; 95% CI = 0.24 to 1.00; within-group *I*^2^ = 0.0%) were compared to PJT interventions with >6 weeks (four study groups; ES = 1.30; 95% CI = 0.41 to 2.20; within-group *I*^2^ = 63.9%). 

Similarly, no significant sub-group difference (between-group *p =* 0.545) was found when PJT interventions with players ≤21.45 years old (five study groups; ES = 1.02; 95% CI = 0.25 to 1.79; within-group *I*^2^ = 65.3%) were compared to PJT interventions with players >21.45 years old (five study groups; ES = 0.75; 95% CI = 0.33 to 1.16; within-group *I*^2^ = 0.0%). 

### 3.8. Meta-Analysis Results for Change of Direction Speed

Five studies provided data for CODS performance, involving eight experimental and five control groups (pooled *n* = 144). There was a significant effect of PJT on CODS performance (ES = 0.73; 95% CI = 0.39 to 1.06; *p <* 0.001; *I*^2^ = 0.0%; Egger’s test *p* = 0.813; Figure 4B). The relative weight of each study in the analysis ranged from 6.3% to 25.8%. 

### 3.9. Meta-Analysis Results for Endurance

**Figure 5:**
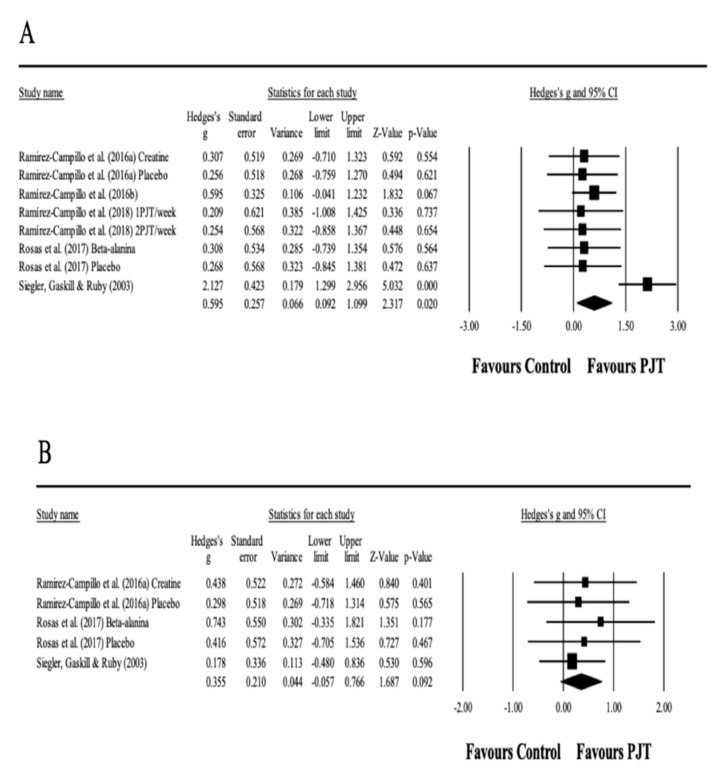
Forest plot of changes in endurance performance (**A**) and anaerobic performance (**B**), in athletes participating in plyometric jump training (PJT) compared to controls. Values shown are effect sizes (Hedges’s g) with 95% confidence intervals (CI). The size of the plotted squares reflects the statistical weight of the study. Split figure and place (**B**) under (**A**) to fit on the page.

Five studies provided data for endurance performance, involving eight experimental and five control groups (pooled *n* = 150). There was a significant effect of PJT on endurance performance (ES = 0.60; 95% CI = 0.09 to 1.10; *p =* 0.020; *I*^2^ = 53.7%; Egger’s test *p* = 0.328; Figure 5A). The relative weight of each study in the analysis ranged from 10.0% to 17.3%. 

### 3.10. Meta-Analysis Results for Anaerobic Performance

Three studies provided data for anaerobic performance, involving five experimental and three control groups (pooled *n* = 89). There was a non-significant effect of PJT on anaerobic performance (ES = 0.36; 95% CI = −0.06 to 0.77; *p =* 0.092; *I*^2^ = 0.0%; Egger’s test *p* = 0.121; Figure 5B). The relative weight of each study in the analysis ranged from 13.5% to 39.2%. Of note, from a pool of 48 potential moderator analyses, due to a limited number of studies (i.e., <3 per moderator), only six moderator analyses were possible (as indicated above). 

### 3.11. Adverse Effects

None of the included studies reported soreness, pain, fatigue, injury, damage, or adverse effects related to the PJT intervention.

## 4. Discussion

Our findings revealed that PJT is effective in improving CMJ and DJ, kicking performance, speed on sprint and COD tests and endurance performance in female soccer players. A discussion of such findings follows below.

### 4.1. Countermovement Jump

A moderate increase in CMJ was observed. The positive effect of PJT on CMJ was previously noted in female athletes [28,35]. Similarly, a meta-analysis in male soccer players revealed also a significant effect of PJT on male athletes’ vertical jump height performance [29]. Although the moderate ES can be considered consistent across the included studies, caution should be taken since one study [47] out of 10 resulted in an extremely large (ES = 4.027) effect, even though the sample was composed by Spanish National Women’s First Division players who are supposed to be highly trained. Similarly, Stojanović et al. [35] showed a large effect in female athletes, but also warned about the presence of one study (out of seven) reporting uncommon large gains (ES = 5.10) in CMJA. In both cases, discrepant effects can inflate results and reveal the necessity of performing more highly controlled studies to reach firm conclusions about the effectiveness of PJT on CMJ with and without arm swing in female soccer players. Nonetheless, after a moderator analysis for the current SRMA, excluding the study that yielded extremely large CMJ improvements [47], a significant improvement in CMJ (ES = 0.48; *p* = 0.002) was still observed. Therefore, PJT seems effective in the improvement of CMJ in female soccer players. Such improvement can be explained by the improved specific neural activation patterns and enhanced SSC utilization after training [17], even though CMJ can be considered a slow SSC movement [53]. 

### 4.2. Countermovement Jump with Arm Swing

Although two out of three studies demonstrated an improvement in CMJA performance, in contrast to CMJ, the CMJA increase was not significant. Although three studies provided data for the current meta-analysis, complying with previous recommendations for robust meta-analysis [54,55,56], the interpretation of results derived from such a limited number of studies should be performed with caution. Firstly, in the study of Ramirez-Campillo et al. [44] and Rubley et al. [46] the participants in the experimental groups had an increase in performance, while the soccer players in the control groups reduced their performance. In contrast, in the study of Siegler et al. [48] the participants from both the PJT and control groups exhibited an improved performance, with a greater improvement observed in the control group, which may explain the negative ES in Figure 2B for the Siegler et al. study [48]. Of note, in the study of Rubley et al. [46], one PJT session was applied each week, in the study of Ramirez-Campillo et al. [44] two sessions per week, whereas in the Siegler et al. study [48] up to three sessions were applied each week. According to a previous meta-analysis in female soccer players, PJT interventions with two or more sessions per week produced a moderate effect on vertical jump height (ES = 0.8), while those with less than two sessions per week produced a large effect (ES = 1.47). Is unlikely that overtraining occurred in the Siegler et al. study [48], since an improvement in performance was observed (similar to the control group). However, the possibility that a reduced frequency of PJT may induce an optimization of the training process, with optimal and efficient training volumes, particularly in female soccer players [50], should be further explored.

The lack of a significant increase in CMJA among female soccer players after PJT is in contrast with the findings from a previous meta-analysis in male adult soccer players, were a significant effect of PJT was noted for vertical jump height [29]. Further, among youth male soccer players a PJT meta-analysis revealed a significant increase in vertical jump height (i.e., CMJ and CMJA) [57]. Future studies may elucidate if different biological mechanisms or if differences in methodological issues between published studies (e.g., training duration; testing procedures) underlay the apparent different responses to PJT between male and female soccer athletes. 

### 4.3. Drop Jump

A moderate improvement was noted for DJ (ES = 0.43; *p* = 0.012). Improvements in DJ (i.e., reactive strength) may be associated with several neuromechanically factors [17,58]. Of particular relevance may be the increased musculotendinous stiffness associated to DJ improvements after PJT [17,59]. Such an increase may be associated with a better running economy [60], particularly at high speeds [61], commonly occurring during female soccer matches [5]. In addition, a better DJ performance may reflect an improved ability to tolerate greater eccentric forces and improved concentric RFD [17,20,62,63] which may contribute to several key soccer-specific movements such as change of direction ability, to better tolerate landings after a jump, and explosive actions such as sprinting capabilities [5,59,64,65]. 

Of note, the improvement in DJ performance in our meta-analysis (ES = 0.43) was lower to that observed in a previous meta-analysis that analyzed the effects of PJT on DJ performance (ES = 0.66) [30]. Moreover, in the aforementioned meta-analyses females improved less their jumping performance (ES = 0.5) compared to males (ES = 0.8) [30]. Moreover, male soccer players showed a large improvement in DJ performance after a PJT intervention (ES=1.6), while female athletes showed only a moderate improvement (ES=0.6) [44]. Independent from the magnitude of the response to PJT in female compared to male soccer players, future studies should elucidate the mechanisms underlying the physical fitness improvements in soccer players after PJT.

### 4.4. Kicking Performance

A very large improvement was noted for kicking performance. Kicking performance is a key ability in soccer, involved in key moments of a match, such as passing actions and scoring goals [5]. Indeed, shooting performance is related with match success and league positioning [66,67,68]. Therefore, improvements in kicking performance after PJT may be important for female soccer players. Such improvement after PJT might be mediated through neuromechanically adaptations, including increased force and rate of force development [17,69,70]. However, from three studies that provided data for kicking performance, one [47] yielded an improvement value that was unusually high (ES = 5.71). Such result may be related to low SD values reported by the authors, probably due to the high performance level (Spanish National Women’s First Division) of the athletes involved in the study. Independent of this, the fact that high-level athletes achieved an improvement in kicking performance of ~Δ12% [47] after PJT should be highlighted. In the study of Rubley et al. [46] a very large improvement was noted (ES = 2.91; Δ22%). Such improvement may not be uncommon among youth players. Indeed, in the study of Rubley et al. [46], youth (age = 13.4 y) athletes were included. In a previous work [71] with youth (male) soccer players (age = 11.8 y), an improvement of ES=1.83 was noted for kicking performance, after a PJT with a comparable load (i.e., 8-16 sessions) as in the study of Rubley et al. [46]. In this sense, soccer skills (e.g., kicking) may be particularly improved at certain age [72], such as in the study by Rubley et al. [46], whereas adult (female) athletes may achieve comparatively less improvements (ES = 0.42–0.45) [50]. When the total volume of PJT was compared between studies, values of 810 jumps [50] 1680 jumps [46] and 3240 jumps were noted. [47]. Whether such volume difference may explain the different magnitude of observed improvements deserves to be examined in future PJT research with female soccer players. Such research may allow to stablish optimal volumes for both increased performance and reduced injury risk [10,73].

### 4.5. Linear Sprint

Linear sprint performance obtained a moderate improvement after PJT. Considering the high frequency of short-distance (<30 m) sprints occurring in soccer matches, improving sprinting ability may increase the probability to winning ball possession and stand out from other players [5,74]. Interestingly, the studies analyzed in this SRMA provided data for 15 m to 20 m sprints (one study provided data for 30 m sprint). Therefore, improvements in linear sprinting probably reflect improved acceleration capabilities [75,76,77]. Considering the relevance of the force-velocity spectrum parameters (i.e., force, power and velocity) during sprinting among soccer players [78], it seems plausible that an optimization of the force-velocity spectrum may help to explain the improvements in sprinting after PJT. Indeed, PJT may provide positive adaptations in the force-velocity spectrum [79,80]. 

Compared to male soccer players, there are some contrast findings in the literature. For example, as in the current meta-analysis, a previous meta-analysis conducted with youth male soccer players observed significant improvements in linear sprint performance after PJT in distances between 5 m, 10 m, 20 m, 30 m, but not 40 m [57]. On the other side, a previous meta-analysis conducted with adult male soccer players observed non-significant improvements in linear sprint performance after PJT in distances between 5, 10, 15 and 30 m, with a significant improvement noted only for 20 m [29]. Future studies may elucidate how sex and maturity might interact to moderate the effects of PJT on linear sprint performance among soccer players. 

### 4.6. Change of Direction Speed

A moderate improvement in COD was observed after PJT. Considering the relevance of COD performance among female soccer players (e.g., 1,336–1,529 movement changes during a match) [81], such improvement may reflect an advantage during competitive matches (e.g., increased goal chances) [2]. Neuromechanically adaptations such as greater muscle activation of the knee flexors (eccentric phase) and extensors (concentric or propulsive phase) [17,82,83] may favor COD improvements [64]. This may allow greater absorption of forces and increase ground reaction force production required in task execution of the COD [84]. Additionally, improvements in plyometric ability may also explain the improved COD capability [85].

The improvement in COD performance among female soccer players is in contrast with the result observed in male soccer players. Indeed, no improvement was noted in COD assessed with the *t* test and the zig-zag test in a previous PJT meta-analysis conducted in male soccer players [29]. Of note, in our meta-analysis female soccer players were assessed for COD performance with the *t* test and the zig-zag test, although also with the Illinois agility test, the latter requiring longer distances. Future studies may elucidate if differences in measurement protocols or sex-related differences might explain the apparently contrast COD performance changes after PJT between male and female soccer players. 

### 4.7. Endurance

Endurance performance obtained a moderate improvement after PJT. Similarly, a meta-analysis revealed a significant effect of PJT on male soccer players endurance capability [29]. Improvements in endurance capacity has shown significant correlations with repeat sprint ability (RSA) [86], the latter positively associated with match-play performance [87]. These improvements could be explained due to better phosphocreatine resynthesize rate [87]. Therefore, increments of absolute strength would result enhancement in economy running due to a reduced recruitment of higher threshold motor units, producing a more economical behavior [88]. Resistance training methods with an emphasis on PJT and eccentric contractions could be advantageous for female soccer players to improve neuromuscular performance such as maximal strength, tendon stiffness and rate of force development.

### 4.8. Anaerobic Performance

Regarding anaerobic performance, most studies addressed the effects of PJT on performance during the repeated-sprint ability (RSA) test, and no meaningful result was obtained while comparing the experimental groups with controls. Though maximal sprinting speed is one of the performance composites of anaerobic performance during repeated sprints [89] other factors affecting recovery between sprints and neuromuscular performance maintenance are key to improve RSA [89,90,91]. Therefore, PJT may have limited potential to improve anaerobic performance, especially when endurance and fatigue-resistance factors need to be changed in order to induce adaptation. However, compared to controls, the PJT groups achieved an ES = 0.36 (*p* = 0.092). This effect probably is related to the significant and moderate effect of PJT on maximal linear sprinting ability in female soccer players. 

Current findings are difficult to compare with those obtained in male soccer players, as most PJT studies that included RSA assessments included youth male soccer players. In one of such studies, youth males (age, 12.7 years; APHV, −1.3), after 8 weeks of jump training, improved (2.1–2.5%; ES = 0.2–1.6) best and total RSA times [92]. Also, in male youth soccer players (age, 13.6 years; Tanner stage II-III) small (0.7–0.8%) improvements were noted in best and mean RSA times after 6 weeks of training [93]. Relatedly, in young adult soccer players (age, 18.4 years), 8 weeks of training induced an improvement (1.4%; ES = 0.85) in mean RSA with COD time (1.4%; ES = 0.85) and related fatigue index (27.8%; ES = 0.91) [94]. Future studies may elucidate if differences in measurement protocols (i.e., RSA vs RSA-COD), players biological maturity or sex-related differences might explain the apparently contrast findings between our results and those previously reported for RSA changes after PJT for male soccer players.

### 4.9. Potential Limitations

Some potential limitations of this SRMA should be acknowledged. Firstly, additional analyses were not always possible as <3 studies were available for at least one of the moderators. Additionally, the use of the median split technique may induce residual confounding and reduced statistical power [95]. Moreover, a meta-regression was not possible due to reduced number of studies available. Further, even though the included studies did not specify any adverse events associated with the PJT intervention, it is unclear if there was an attempt by the researchers to comprehensively record all possible adverse events. Therefore, future studies are encouraged to describe with more detailed data about possible injuries, pain and/ or any other potential adverse effects, as this would expand our knowledge on the safety of PJT. Finally, only two of the included studies recruited youth female soccer players, and their biological maturity was not described. Considering the potential of biological maturity as a moderator of the effects of PJT on youth female physical fitness adaptations [96,97,98], future studies should strive for the inclusion of biological maturity description among youth female soccer players. 

### 4.10. Practical Applications Derived from the Systematic Review

According to the results of our meta-analysis female soccer players should incorporate PJT programs into their regular training schedules in order to achieve small-large improvements in several physical fitness measures of key relevance in soccer, including jumping, sprinting, kicking, change of direction speed, and endurance. According to our systematic review results, PJT is effective in both youth and adult female soccer players, (age range: 13–27 years), with or without previous experience in PJT, from amateur to professional level. The PJT among the included studies in this meta-analysis proved to be safe, with no injuries reported. Indeed, PJT may be considered an integral part of neuromuscular training programs focused on injury prevention [9,99].

Regarding the characteristics of effective PJT interventions, it seems that a training frequency of 1-3 sessions per week, during 6–12 weeks, with a maximal- near-maximal intensity, is an adequate stimulus to boost physical fitness. Most studies incorporated some form of drop-like jump, although all the studies included different types of jump drills into their programs. The total number of jumps range between 800 up to 5620 jumps. Caution is warranted when high volume of PJT is prescribed. Indeed, a high volume of PJT may increase the injury risk among female soccer players [73]. As a moderate volume of PJT may be as effective compared to a program with a greater volume [50,100] a moderate-volume of PJT is advised, particularly during initial stages of PJT, in those unexperienced with PJT, poor technical ability, and reduced ability to cope with the eccentric forces associated to jumping drills. In line with the volume of PJT, most studies in the meta-analysis did not incorporated a taper. Such strategy may allow to boost performance before important competitions, thus its incorporation may be adequate, particularly with a high-volume PJT program [101].

Rest between sets range from 30 s up to 300 s, and for inter-repetition rest values from 5 up to 60 s were noticed. As 30 s and 120 s of inter-set rest are equally effective in order to allow significant physical fitness improvements after PJT among soccer players [102], and 15 s of inter-repetition rest is adequate to recover between maximal jumping efforts [103], in order to reduce the total duration of a PJT session, coaches may consider values from the low spectrum of above mentioned rest intervals. The minimum inter-session rest was 48 h among the included studies, which seems a common an adequate minimal recovery time between sessions.

A grass surface was the most common type of surface used among the included studies, which seems to be in line with the training principle of specificity, as soccer players usually train and compete on this type of surface. All the studies included in this meta-analysis incorporated a progressive overload, either in the form of volume, type of drill (e.g., two leg, progressing toward one leg), intensity, or a combination of these. Of note, most studies incorporated PJT during the in-season period, demonstrating that during such period of the season significant improvement in relevant physical fitness measures are possible among female soccer players, which may boost performance during important competitive match dates, thus increasing chances to achieve a better league positioning [4].

## 5. Conclusions

Several key physical fitness traits for female soccer players may be improved after PJT, including CMJ, DJ, kicking performance, linear speed, COD speed, and aerobic endurance performance, without changing anaerobic performance. Such improvements may be expected after PJT interventions with 6 or more weeks of duration, and among players with different chronological age, competitive level and soccer experience.

## Figures and Tables

**Table 1:** Characteristics of included study participants and of PJT programs.

Authors	Treat	Age (y) *	SPT	Fit	Freq	Dur	Int	BH (cm)	NTJ	Tply
Chimera et al. 2004	WD	20.0	No	Moderate-high	2	6	Max (RSI; quickness)	45	3940 + 1680 s	B + V + C + A + L + Turn
Fischetti et al. 2019	WD	26.6	Yes	High	3	12	Max (height; distance; velocity; RSI)	60 (hurdle) 50 (stands)	3240	B + C + V + H
Ozbar et al. 2014	WD	18.3	Yes	Moderate	1	8	NR	20 to 60 (hurdle)	1210	H + V + L + D + U + B + A + C
Ramirez-Campillo et al. 2018	WD	22.1	No	Moderate	1-2	8	Max (height; distance, RSI)	5 to 35 (optimal (RSI)	810	U + B + C + A + V + H + Turn + fast SSC + slow SSC
Ramirez-Campillo et al. 2016a	WD	23	No	Moderate	2	6	Max (height; distance; RSI)	20	1440	V + H + U + B + C + A
Ramirez-Campillo et al. 2016b	WD	22.4	No	Moderate-high	2	6	Max (height; distance; RSI)	NA	1440	U + B + V + H + C + A
Rosas et al. 2017	WD	23.6	No	Normal	2	6	Max (height; distance; RSI)	40	1440	V + H + U + B + C + A
Rubley et al. 2011	ID	13.4	No	Moderate-high	1	12	NR	30	1680	U + H + B + V + L + A + C
Sedano et al. 2009	WD	22.8	Yes	High	3	12	Max (height; distance; velocity; RSI)	50 to 60	3240	V + H + B + C + A
Siegler et al. 2003	ID	16.5	No	Normal-moderate	1–3	10	Max (height; distance; quickness)	18 to 42	1046 + 70 s + 900 m	B + V + A + C + H + U + L + Back + D

Note: abbreviations descriptions ordered alphabetically. *: mean values reported for experimental and control groups; A: acyclical (non-repeated); B: bilateral; C: cyclical (repeated); D: diagonal; BH: box height (for those drills that required the use of a box, not necessarily applied to drop jumps); Com: combined PJT with another type of drill; Fitness level: classified as in the recent review by Ramirez-Campillo et al. [28], (i) NR, (ii) high encompasses professional/elite athletes with regular enrolment in national and/or international competitions or highly trained participants with ≥10 training hours per week or ≥6 training sessions per week and a regularly scheduled official or friendly competition, (iii) moderate encompasses non-elite/professional athletes, with a regular attendance in regional and/or national competitions, between 5–9.9 training hours per week or 3–5 training sessions per week and a regularly scheduled official or friendly competition, (iv) normal encompasses recreational athletes with <5 training hours per week with sporadic or no participation in competition; Freq: PJT frequency (sessions per week); H: horizontal; I: intensity; ID: insufficiently described, when the PJT treatment description omitted the reporting of any of the following: duration, frequency, intensity, type of exercises, sets, repetitions; IS: in-season; L: lateral; Max: maximal, involving either maximal effort to achieve maximal height, distance, RSI, velocity, or another marker of intensity; NA: non-applicable; NR: non-reported; NTJ: number of total jumps; PJT: plyometric jump training; PO: progressive overload, in the form of either volume, intensity, or a combination of these; RBR: rest time between repetitions (only when the PJT programs incorporated non-repeated jumps); RBS: rest time between sets; RBTS: rest between training sessions; Rep: replace, denoting if the athletes replace some common drills with PJT drills; RSI: reactive strength index; RT: resistance training; SPT: indicates if the participants had previous systematic experience with PJT; SSC: stretch-shortening cycle; Surf: type of surface used during the intervention; T: type of drill; TP: training period; Tply: type of PJT drills used; U: unilateral; V: vertical; Vo: volume; WD: well described, when treatment description allowed for adequate study PJT replication, including the reporting of duration, frequency, intensity, type of exercises, sets, and repetitions.

**Table 2:** Characteristics of PJT programs.

Authors	Com	RBS (s)	RBR (s)	RBTS (Hours)	Tsurf	PO	TP	Replace	Taper
Chimera et al. 2004	No	30 to 120	NR	NR	NR	Vo + T	OS	A	No
Fischetti et al. 2019	No	240	30 to 60	48 to 72	Hard synthetic floor	Vo	IS	Yes	Yes
Ozbar et al. 2014	No	60 to 300	NR	168	NR	Vo + I + T	IS	No	No
Ramirez-Campillo et al. 2018	No	30 to 60	5 to 15	48 to 168	Combined (grass, land, dirt)	Vo	IS	Yes (6%)	Yes
Ramirez-Campillo et al. 2016a	No	60	15	48 or more	Grass	Vo	IS	Yes	No
Ramirez-Campillo et al. 2016b	No	60	15	72 or more	Grass	Vo	IS	Yes	No
Rosas et al. 2017	No	60	15	48 or more	Grass	Vo	IS	Yes	No
Rubley et al. 2011	Cutting drills	NR	NR	168	NR	T	IS	No	No
Sedano et al. 2009	No	30 to 300	NR	48 to 72	Hard synthetic floor	Vo	IS	Yes	Yes
Siegler et al. 2003	RT + sprints	NR	NR	48 to 144	Grass	Vo + T	IS	Yes	Yes

Note: abbreviations descriptions ordered alphabetically. *: mean values reported for experimental and control groups; A: acyclical (non-repeated); B: bilateral; C: cyclical (repeated); D: diagonal; BH: box height (for those drills that required the use of a box, not necessarily applied to drop jumps); Com: combined PJT with another type of drill; Fitness level: classified as in the recent review by Ramirez-Campillo et al. [28], (i) NR, (ii) high encompasses professional/elite athletes with regular enrolment in national and/or international competitions or highly trained participants with ≥10 training hours per week or ≥6 training sessions per week and a regularly scheduled official or friendly competition, (iii) moderate encompasses non-elite/professional athletes, with a regular attendance in regional and/or national competitions, between 5–9.9 training hours per week or 3–5 training sessions per week and a regularly scheduled official or friendly competition, (iv) normal encompasses recreational athletes with <5 training hours per week with sporadic or no participation in competition; Freq: PJT frequency (sessions per week); H: horizontal; I: intensity; ID: insufficiently described, when the PJT treatment description omitted the reporting of any of the following: duration, frequency, intensity, type of exercises, sets, repetitions; IS: in-season; L: lateral; Max: maximal, involving either maximal effort to achieve maximal height, distance, RSI, velocity, or another marker of intensity; NA: non-applicable; NR: non-reported; NTJ: number of total jumps; PJT: plyometric jump training; PO: progressive overload, in the form of either volume, intensity, or a combination of these; RBR: rest time between repetitions (only when the PJT programs incorporated non-repeated jumps); RBS: rest time between sets; RBTS: rest between training sessions; Rep: replace, denoting if the athletes replace some common drills with PJT drills; RSI: reactive strength index; RT: resistance training; SPT: indicates if the participants had previous systematic experience with PJT; SSC: stretch-shortening cycle; Surf: type of surface used during the intervention; T: type of drill; TP: training period; Tply: type of PJT drills used; U: unilateral; V: vertical; Vo: volume; WD: well described, when treatment description allowed for adequate study PJT replication, including the reporting of duration, frequency, intensity, type of exercises, sets, and repetitions.

**Table 3:** Study groups and their physical fitness.

		PJT, Before ^α^			Control, Before			PJT, After			Control, After		
Author (Year)	Test	Mean	SD	*n*	Mean	SD	*n*	Mean	SD	*n*	Mean	SD	*n*
Chimera et al. 2004	Jump (DJ 45-cm; cm)	17.89	2.29	9	18.17	2.24	9	18.89	2.45	9	18.5	2.06	9
Fischetti et al. 2019	Jump (CMJ; cm)	33.6	5.5	14	32.1	6.4	14	36.8	5.8	14	32.7	5.7	14
	CODS (T-test; s)	8.8	0.3	14	8.9	0.3	14	8.5	0.3	14	8.8	0.4	14
Ozbar et al. 2014	Jump (CMJ; cm)	39.8	4.5	9	35.4	4.6	9	46.8	2.2	9	37.9	3.9	9
	Vertical jump power (CMJ; w)	3480	643.2	9	2492.2	432.1	9	3855.2	536.6	9	3080.2	420.4	9
	Linear sprint (20 m; s)	3.7	0.3	9	3.9	0.4	9	3.4	0.2	9	4	0.5	9
Ramírez-Campillo et al. 2018 (1 PJT/week)	Jump (CMJ; cm)	28.5	6.9	8	28.8	4.9	3	31.5	7.5	8	29.9	5.1	3
	Jump (DJ 20 cm; cm)	27.2	5.9	8	28.7	4.3	3	30.9	7.8	8	29.3	4.6	3
	Kicking ability (Instep kick; km·h^−1^)	65.1	9	8	67.3	7.2	3	70.6	8.9	8	68.9	7.5	3
	Linear sprint (15 m; s)	3.28	0.1	8	3.42	0.2	3	3.01	0.1	8	3.45	0.2	3
	CODS (Meylan test; s)	4.94	0.2	8	4.96	0.2	3	4.57	0.2	8	4.95	0.4	3
	Endurance (Yo-yo test level 1; m)	573	237	8	606	175	3	628	224	8	612	179	3
Ramírez-Campillo et al. 2018 (2 PJT/week)	Jump (CMJ; cm)	27.4	4.3	8	28.8	4.9	4	30.1	4.7	8	29.9	5.1	4
	Jump (DJ 20 cm; cm)	27.7	5.8	8	28.7	4.3	4	31.3	6.6	8	29.3	4.6	4
	Kicking ability (instep kick; km·h^−1^)	63	9.5	8	67.3	7.2	4	68.9	11	8	68.9	7.5	4
	Linear sprint (15 m; s)	3.43	0.1	8	3.42	0.2	4	3.1	0.1	8	3.45	0.2	4
	CODS (Meylan test; s)	5.12	0.3	8	4.96	0.2	4	4.74	0.3	8	4.95	0.4	4
	Endurance (Yo-yo Test IR1; m)	630	192	8	606	175	4	690	203	8	612	179	4
Ramírez-Campillo et al. 2016a (placebo)	Jump (CMJ; cm)	28.7	5.1	10	25.9	4.1	5	30	5.3	10	25.9	3.2	5
	Vertical jump power (CMJ; w)	1940	338	10	1979	211	5	2037	354	10	1914	249	5
	Jump (DJ; mm·mm^−1^)	1.36	0.4	10	1.4	0.66	5	1.36	0.4	10	1.39	0.6	5
	Linear sprint (20 m; s)	3.87	0.3	10	3.99	0.2	5	3.74	0.26	10	3.98	0.14	5
	CODS (Illinois test; s)	18.8	1.2	10	19.4	0.8	5	18.2	0.9	10	19.3	0.5	5
	Endurance (20 m mult-stage shuttle run test; min)	7.8	1.5	10	7.4	1.9	5	8.3	1.3	10	7.5	1.8	5
	Anaerobic performance (Test RAST mean; s)	7.08	0.6	10	7.35	0.5	5	6.78	0.53	10	7.2	0.31	5
Ramírez-Campillo et al. 2016a (creatine)	Jump (CMJ; cm)	27.3	5.2	10	25.9	4.1	5	28.9	4.6	10	25.9	3.2	5
	Vertical Jump Power (CMJ; w)	1969	250	10	1979	211	5	2108	278	10	1914	249	5
	Jump (DJ; mm·mm^−1^)	1.33	0.3	10	1.4	0.6	5	1.46	0.3	10	1.39	0.6	5
	Linear sprint (20 m; s)	3.98	0.4	10	3.99	0.2	5	3.85	0.37	10	3.98	0.14	5
	CODS (Illinois test; s)	19.3	1.1	10	19.4	0.8	5	18.8	0.8	10	19.3	0.5	5
	Endurance (20 m mult-stage shuttle run test; min)	8	1.6	10	7.4	1.9	5	8.5	1.3	10	7.5	1.8	5
	Anaerobic performance (RAST mean; s)	7.48	1	10	7.35	0.5	5	7.08	0.88	10	7.3	0.31	5
Ramírez-Campillo et al. 2016b	Jump (CMJ; cm)	26.7	5.5	19	26.6	4.8	19	29.4	5.8	19	26.6	4.3	19
	Jump (CMJA; cm)	30.3	6.5	19	29.2	5.5	19	32.6	6.5	19	28.9	5.1	19
	Jump (DJ 40 cm; cm·ms^−1^)	0.119	0.04	19	0.101	0.03	19	0.144	0.04	19	0.107	0.03	19
	Linear sprint (30 m; s)	5.69	0.31	19	5.72	0.28	19	5.4	0.32	19	5.82	0.31	19
	CODS (Illinois test; s)	19.48	0.9	19	19.79	1	19	18.73	1	19	19.93	0.9	19
	Endurance (20 m mult-stage shuttle run test; min)	8.4	1.9	19	8.6	1.6	19	9.1	1.2	19	8.6	1.1	19
Rosas et al. 2017 (placebo)	Jump (CMJ; cm)	24.8	3.4	8	28.9	5.8	5	26.4	3	8	29.4	6.3	5
	Vertical jump power (CMJ; w)	1974	259	8	2003	341	5	2140	250	8	1989	272	5
	Jump (DJ 40 cm; cm)	1.24	0.4	8	1.33	0.3	4	1.67	0.6	8	1.33	0.5	4
	Linear sprint (20 m; s)	3.89	0.4	8	3.78	0.3	4	3.77	0.4	8	3.83	0.4	4
	CODS (Illinois test; s)	18.5	0.3	8	18.7	0.4	4	18.2	0.4	8	18.7	0.4	4
	Endurance (20 m shuttle run; min)	7.1	1.1	8	7.9	1.8	4	7.5	1	8	7.9	2	4
	Anaerobic performance (RAST mean; s)	7.49	0.8	8	7.18	0.9	4	7.18	0.6	8	7.17	0.8	4
Rosas et al. 2017 (beta-alanina)	Jump (CMJ; cm)	28.1	3.5	8	28.9	5.8	4	30.6	3.1	8	29.4	6.3	4
	Vertical Jump Power (CMJ; w)	1944	340	8	2003	341	4	2122	318	8	1989	272	4
	Jump (DJ 40 cm; cm)	1.11	0.2	8	1.33	0.3	5	1.53	0.5	8	1.33	0.5	5
	Linear sprint (20 m; s)	3.92	0.2	8	3.78	0.3	5	3.8	0.1	8	3.83	0.4	5
	CODS (Illinois test; s)	18.9	0.7	8	18.7	0.4	5	18.6	0.8	8	18.7	0.4	5
	Endurance (20 m shuttle-run; min)	7.9	1.7	8	7.9	1.8	5	8.5	1.7	8	7.9	2	5
	Anaerobic performance (RAST mean; s)	7.61	0.5	8	7.18	0.9	5	7.1	0.5	8	7.17	0.8	5
Rubley et al. 2011	Jump (CMJA; cm)	39.6	8.2	10	39.4	8.3	6	47	8.1	10	39.6	8.2	10
	Kicking ability (m)	25.9	2.6	10	27.6	2.5	6	33	3.7	10	23.3	3.7	6
Siegler et al. 2003	Jump (CMJA; cm)	37.65	0.15	17	36.46	3.68	17	39.37	4.69	17	39.19	4.45	17
	Vertical jump power (Wingate 30 s; kg·m·min^−1^)	10.36	2.38	17	9.59	0.92	17	10.68	2.2	17	9.78	1.36	17
	Linear sprint (20 m; s)	3	0.15	17	2.89	0.13	17	2.9	0.13	17	2.85	0.13	17
	Endurance (LIST; s)	646	167.5	17	1064	195.2	17	1040	157.33	17	1115	157.51	17
	Anaerobic performance (Wingate 30 s; kg·m·min^−1^)	7.27	0.49	17	7.76	0.6	17	7.37	0.64	17	7.73	0.78	17
Sedano-Campo et al. 2009	Jump (CMJ; cm)	25.6	1	10	26.2	0.9	10	29.3	1	10	25.9	0.9	10
	Jump (DJ 40 cm; cm)	24.9	1.1	10	27.1	1	10	28.9	0.8	10	25.6	0.9	10
	Kicking ability (km·h^−1^)	70	2.4	10	75.8	1.5	10	78.3	2.1	10	74.1	1.1	10

^α^: before and after values denotes the mean ± standard deviation for each group before and after the intervention, respectively. Note: abbreviations descriptions ordered alphabetically. CMJ: countermovement jump; CMJA: countermovement jump with arm swing; CODS: change of direction speed; DJ: drop jump; LIST: Loughborough intermittent shuttle test; RAST: repeated anaerobic sprint test; SD: standard deviation.

**Table 4:** Physiotherapy Evidence Database (PEDro) scale ratings.

	N° 1 *	N° 2	N° 3	N° 4	N° 5	N° 6	N° 7	N° 8	N° 9	N° 10	N° 11	Total **
Chimera et al. 2004	1	1	0	1	0	0	0	1	0	1	1	5
Fischetti et al. 2019	1	1	0	1	0	0	0	1	1	1	1	6
Ozbar et al. 2014	0	1	0	1	0	0	0	1	0	1	1	5
Ramirez-Campillo et al. 2018	1	1	1	1	0	0	1	0	0	1	1	6
Ramirez-Campillo et al. 2016 a	0	1	1	1	0	1	1	1	0	1	1	9
Ramirez-Campillo et al. 2016 b	0	1	1	1	0	0	0	1	0	1	1	7
Rosas et al. 2018	0	1	1	1	1	1	1	1	0	1	1	9
Rubley et al. 2011	0	0	0	1	0	0	0	1	0	1	1	4
Sedano-Campo et al. 2009	0	1	0	1	0	0	0	1	0	1	1	5
Siegler et al. 2003	0	0	0	1	0	0	0	1	0	1	1	4

*: PEDro scale items number; **: the total number of points from a possible maximal of 10. A detailed explanation for each PEDro scale item can be accessed at https://www.pedro.org.au/english/downloads/pedro-scale. In brief: item 1, eligibility criteria were specified; item 2, participants were randomly allocated to groups; item 3, allocation was concealed; item 4, the groups were similar at baseline; item 5, there was blinding of all participants regarding the plyometric jump training programme being applied; item 6, there was blinding of all coaches responsible for the application of plyometric jump training programme regarding its aim toward the improvement of physical fitness; item 7, there was blinding of all assessors involved in measurement of physical fitness attributes; item 8, measures of at least one key fitness variable were obtained from more than 85% of participants initially allocated to groups; item 9, all participants for whom fitness variables were available received the treatment or control condition as allocated or, data for at least one key fitness variable was analysed by “intention to treat”; item 10, the results of between-group statistical comparisons are reported for at least one key fitness variable; and item 11, point measures and measures of variability for at least one key fitness variable are provided.
